# Insights into the metabolic response to traumatic brain injury as revealed by ^13^C NMR spectroscopy

**DOI:** 10.3389/fnene.2013.00008

**Published:** 2013-10-04

**Authors:** Brenda L. Bartnik-Olson, Neil G. Harris, Katsunori Shijo, Richard L. Sutton

**Affiliations:** ^1^Department of Radiology, Loma Linda University School of MedicineLoma Linda, CA, USA; ^2^Department of Neurosurgery, David Geffen School of Medicine at University of California Los AngelesLos Angeles, CA, USA

**Keywords:** acetate, glucose, glutamate-glutamine cycle, magnetic resonance spectroscopy, neuroglial compartmentation, oxidative metabolism, pentose phosphate pathway

## Abstract

The present review highlights critical issues related to cerebral metabolism following traumatic brain injury (TBI) and the use of ^13^C labeled substrates and nuclear magnetic resonance (NMR) spectroscopy to study these changes. First we address some pathophysiologic factors contributing to metabolic dysfunction following TBI. We then examine how ^13^C NMR spectroscopy strategies have been used to investigate energy metabolism, neurotransmission, the intracellular redox state, and neuroglial compartmentation following injury. ^13^C NMR spectroscopy studies of brain extracts from animal models of TBI have revealed enhanced glycolytic production of lactate, evidence of pentose phosphate pathway (PPP) activation, and alterations in neuronal and astrocyte oxidative metabolism that are dependent on injury severity. Differential incorporation of label into glutamate and glutamine from ^13^C labeled glucose or acetate also suggest TBI-induced adaptations to the glutamate-glutamine cycle.

## Introduction

A significant body of work has shown that traumatic brain injury (TBI) initiates a cascade of cellular events including potassium efflux (Katayama et al., 1990; Kawamata et al., 1995), Ca^++^ accumulation (Fineman et al., 1993; Osteen et al., 2001), glutamate release (Katayama et al., 1990; Nilsson et al., 1990; Rose et al., 2002), and increased oxidative stress (Hall et al., 1993; Lewen and Hillered, 1998; Vagnozzi et al., 1999; Tyurin et al., 2000; Marklund et al., 2001) that contribute to reduced ATP production. In addition, TBI results in an immediate increase in cerebral metabolic rates for glucose (CMRglc) (Yoshino et al., 1991; Sutton et al., 1994; Lee et al., 1999; Kelly et al., 2000) that can endure for days in TBI patients (Bergsneider et al., 1997). This increase is thought to represent an increase in glycolysis (hyperglycolysis) in an attempt to meet the cellular energy demand required to restore ionic balance and maintain the neuronal membrane potential (Hovda, 1996). Studies have shown that the duration and severity of regional decreases in ATP are dependent upon TBI severity (Lee et al., 1999; Aoyama et al., 2008; Signoretti et al., 2010), and during the post-injury period where ATP production is reduced, secondary insults or activation of the injured brain can further reduce ATP levels and result in secondary cellular damage (Ip et al., 2003; Zanier et al., 2003; Aoyama et al., 2008). A secondary and enduring reduction of CMRglc (metabolic “depression”) is a common finding in models of experimental TBI (Hovda et al., 1991; Yoshino et al., 1991; Sutton et al., 1994; Jiang et al., 2000; Moore et al., 2000; Prins and Hovda, 2001) and after human TBI (Langfitt et al., 1986; Yamaki et al., 1996; Bergsneider et al., 2000, 2001). Moreover, an increase in energy demand or decreased glucose availability after TBI would potentially compromise neuronal viability and functional outcomes (Vespa et al., 2003, 2007; Parkin et al., 2005; Marcoux et al., 2008).

## Potential factors contributing to the metabolic depression after TBI

Some underlying mechanisms responsible for the hypometabolic response following TBI are the overproduction of reactive oxygen and nitrogen species which can lead to poly(ADP) ribose polymerases (PARP) activation (Laplaca et al., 1999; Clark et al., 2001; Arundine et al., 2004; Mendez et al., 2004; Kauppinen, 2007; Besson, 2009) and related reductions of nicotinamide adenine dinucleotide (NAD^+^) and nicotinamide adenine dinucleotide phosphate (NADP^+^; Satchell et al., 2003; Clark et al., 2007; Signoretti et al., 2010). Consequently, a diminished supply of reducing equivalents for oxidoreductive reactions involved in glucose metabolism, such as glyceraldehyde-3-phosphate dehydrogenase (GAPDH) and pyruvate dehydrogenase (PDH), could inhibit glycolysis and the entry of pyruvate into the TCA cycle resulting in energy depletion and cell death. Reduced GAPDH activity has also been shown to act as a “molecular switch” resulting in increased flux of glucose into the PPP (Ralser et al., 2007; Grant, 2008). Direct evidence for reduced activity of these enzyme complexes following TBI is an active area of research, and to date, studies have shown PDH nitrosylation (Opii et al., 2007) and alterations in the expression and phosphorylation of PDH E1alpha1 subunit (Sharma et al., 2009; Xing et al., 2009, 2012). Both increased intracellular Ca^++^ or PARP activity (Lai et al., 2008) following TBI can also lead to an uncoupling of the mitochondrial electron transport chain (Dugan et al., 1995) and mitochondrial permeability transition (Gunter et al., 1994; Schinder et al., 1996; Zamzami et al., 1997), with decreases in state 3 respiratory rates (Xiong et al., 1997, 1998; Verweij et al., 2000) that would contribute to energy loss and cell death. Studies have shown that PARP inhibitors can attenuate NAD^+^ reductions, decrease neuronal damage, and improve behavioral outcome following experimental TBI (Laplaca et al., 2001; Komjati et al., 2005; Clark et al., 2007; Besson, 2009).

## ^13^C studies of TBI

**Figure 1:**
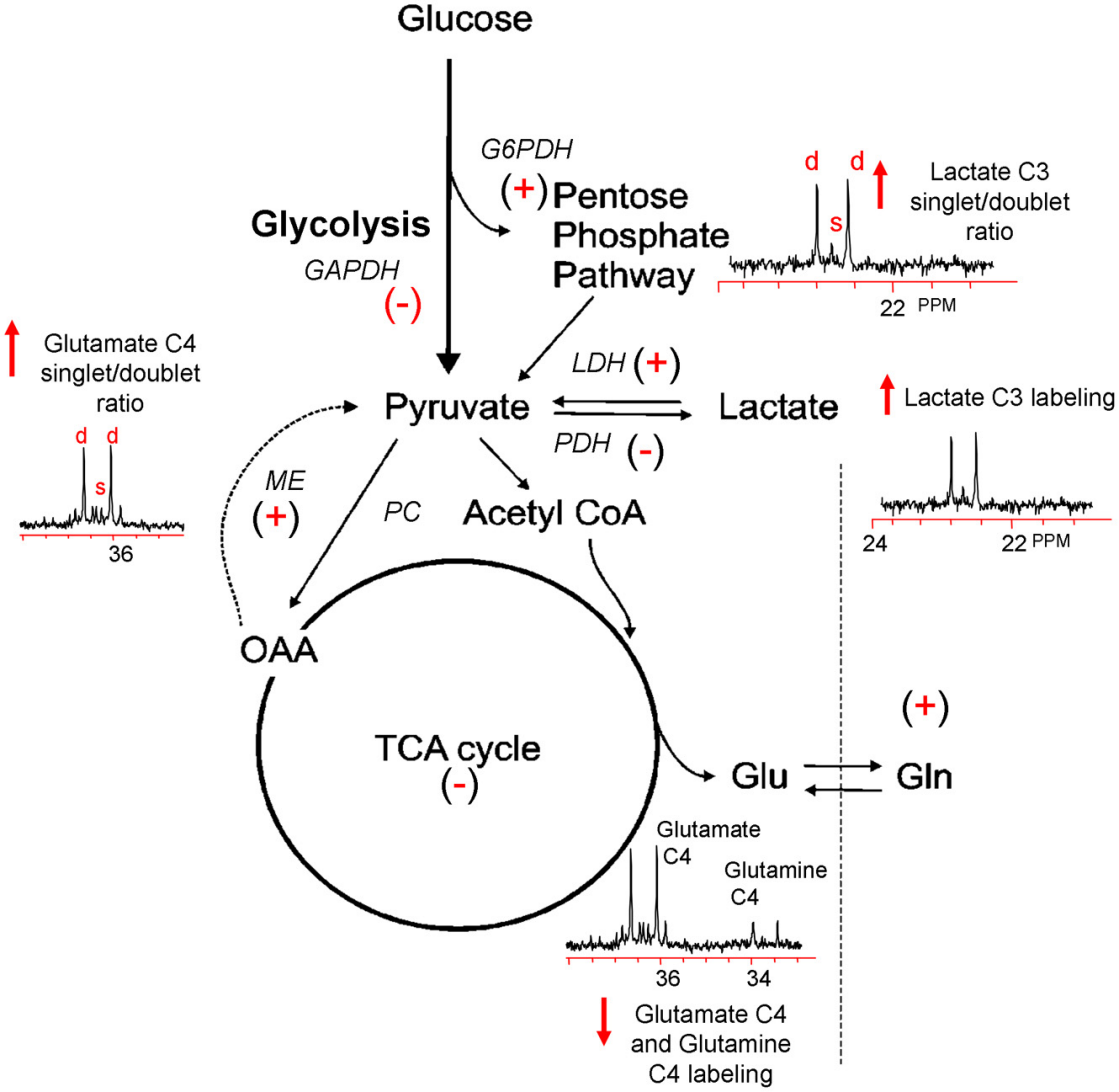
**Simplified illustration of the metabolic response to TBI as determined by ^13^C NMR spectroscopy using [1, 2 ^13^C_2_] glucose.** TBI-induced ion fluxes and neurotransmitter release can increase anaerobic metabolism and initiate an injury cascade including increased oxidative/nitrosative stress, PARP-1 activation, and NAD^+^ and/or NAPD^+^ reductions. These in turn are thought to result in the activation (^+^) or inhibition (−) of enzymes contributing to the metabolic response to TBI (see text). Using [1, 2 ^13^C_2_] glucose as a precursor, ^13^C NMR spectroscopy can be used to measure increases (↑) or decreases (↓) in glycolysis, the PPP, oxidative metabolism in the TCA cycle, and the pyruvate recycling system by monitoring the production of [2, 3 ^13^C_2_] lactate, [2 ^13^C] lactate, and [4, 5 ^13^C_2_] glutamate and [4 ^13^C] glutamate.

To more finely resolve the TBI-induced changes in glucose metabolic pathways, a number of studies have employed the use of stable isotopes (^13^C) of glucose, lactate and acetate to determine the metabolic fate of these fuels and characterize changes in oxidative metabolism and neuroglia metabolic compartmentation during the acute and hypometabolic periods following experimental and clinical TBI (Bartnik et al., 2005, 2007; Dusick et al., 2007; Gallagher et al., 2009; Scafidi et al., 2009; Bartnik-Olson et al., 2010; Clausen et al., 2011). In most studies these isotopes were used in conjunction with *ex vivo* ^13^C nuclear magnetic resonance (NMR) spectroscopy, which allows for the simultaneous assessment of multiple metabolic pathways. The primary advantage of this technique arises from its ability to distinguish ^13^C incorporation into multiple metabolites as well as into the specific carbon positions within the same metabolite, resulting in a detailed analysis of the metabolic “fate” of the ^13^C label (Bachelard and Badar-Goffer, 1993; Cruz and Cerdan, 1999). The relative ^13^C enrichment at each carbon position and the ratios between isotopomers of glutamate and glutamine gives additional information regarding enzyme usage, neurotransmitter synthesis, and neuroglia metabolic compartmentation (Badar-Goffer et al., 1990; Shank et al., 1993; Hassel et al., 1995; Aureli et al., 1997). As shown in Figure 1, ^13^C NMR spectroscopy can be used to measure TBI-induced changes in glycolysis (^13^C lactate labeling), oxidative metabolism within, and interactions between, the neuron and astrocyte compartments (glutamate and glutamine labeling). By using [1, 2 ^13^C_2_] glucose as the substrate, injury-induced changes in the activity of the pentose phosphate pathway (PPP) can be assessed using the ratio between the lactate labeled in two carbon positions (doublet) via glycolysis (C2 and C3) and lactate labeled in the singlet C3 carbon position (Cruz et al., 1998; Lee et al., 1998). However, the formation of a lactate C3 singlet also results via the pyruvate recycling pathway (Hassel and Sonnewald, 1995). In contrast, lactate labeling using [1, 2 ^13^C_2_] acetate is derived exclusively from the pyruvate recycling pathway and comparisons between the lactate C3 singlet/doublet ratio from [1, 2 ^13^C_2_] glucose and the lactate labeling from [1, 2 ^13^C_2_] acetate could provide a valid method for more accurately measuring the contribution of the PPP to the post-injury response. The cytosolic NAD^+^/NADH redox state of the injured tissue can be estimated by the ratio of glutamate labeled in two carbon positions via glycolysis (C3 and C4) to the labeling of glutamate labeled in the C4 position via the recycling of pyruvate from labeled oxaloacetate via malic enzyme using either [1, 2 ^13^C_2_] glucose or [1, 2 ^13^C_2_] acetate (ME; Cruz et al., 1998; Cerdan et al., 2006). This more finely-grained approach to monitoring the fate of labeled fuels provides an ideal platform from which to determine how fuel supplementation after injury may prevent on-going metabolic deficits.

### Experimental models used in ^13^C studies of TBI

To date ^13^C studies have been conducted using the lateral fluid percussion injury (FPI) or the unilateral controlled cortical impact (CCI) injury models of TBI. The FPI model can produce a diffuse pattern of neuronal and axonal injury, while CCI is often termed a more focal injury model due to the contusion induced (Cernak, 2005) although widespread axonal injury occurs after CCI (Hall et al., 2008). With moderate injury severity the period of hyperglycolysis and the extent of ATP reduction in cortex is reduced in the FPI model compared to CCI (Lee et al., 1999), but in both TBI models a widespread reduction in CMRglc is observed throughout the injured hemisphere within a few hours (Hovda et al., 1991; Yoshino et al., 1991; Sutton et al., 1994; Moore et al., 2000). Although CMRglc recovers to baseline within 10 days after FPI (Yoshino et al., 1991; Moore et al., 2000) substantial reductions of CMRglc are still present by 15 days following CCI injury (Moro et al., 2011).

### Glycolysis and PPP metabolism following TBI

Numerous experimental studies have reported an acute increase in extracellular lactate levels consistent with hyperglycolysis following TBI (Inao et al., 1988; Kawamata et al., 1995; Chen et al., 2000). In keeping with these observations, studies using *ex vivo* ^13^C NMR spectroscopy have reported increased ^13^C labeling of lactate from ^13^C glucose in the injured cortex within the first 6 h following a CCI injury in both the adult (Bartnik et al., 2005) and immature rat brain (Scafidi et al., 2009). In the adult brain this increase was seen at 3.5 h after injury which then normalized by 24 h (Bartnik et al., 2005). Increased lactate labeling from ^13^C-labeled glucose was also detected by gas chromatography/mass spectroscopy (GC-MS) from microdialysis samples of CCI injured cerebral cortex in rats (Clausen et al., 2011) and in blood samples from moderate-severe human TBI patients (Dusick et al., 2007). In adult rats with FPI, lactate ^13^C labeling was reduced at 24 h post-injury (Bartnik et al., 2007) indicating reduced glucose metabolism via glycolysis in this more diffuse TBI model. It remains to be determined whether this finding is the result of reduced GAPDH activity and whether similar findings are replicated at all developmental stages.

*Ex vivo* ^13^C NMR spectroscopy studies of cerebral cortex after CCI or FPI using [1, 2 ^13^C_2_] glucose also reported a significant increase in lactate labeling via the PPP (Bartnik et al., 2005, 2007). This finding was supported by a clinical study of severe TBI patients, where evidence of increased glucose metabolism via the PPP was detected in blood samples using GC-MS following an infusion of [1, 2 ^13^C_2_] glucose (Dusick et al., 2007). The PPP functions in producing reducing equivalents of NADPH for biosynthetic reactions (Baquer et al., 1988) and it has been demonstrated that this pathway has an enormous reserve capacity that can be drawn on during periods of oxidative stress to act as a proton donor during the redox cycling of glutathione (Hothersall et al., 1982; Schrader et al., 1993). Peroxynitrite has been shown to activate glucose-6-phosphate dehydrogenase (G6PDH), the enzyme catalyzing the rate limiting step of the oxidative branch of the PPP, resulting in the rapid activation of the PPP and increased NADPH accumulation in astrocytes and neurons (Garcia-Nogales et al., 2003). In addition, *in vitro* studies of neurons and astrocytes in high glucose environments show increased PPP activity and glutathione levels in astrocytes, which can reduce levels of oxidative stress and protect neurons in mixed cultures (Takahashi et al., 2012). Recently, it was shown that pyruvate generated from metabolism via the PPP can be metabolized in the TCA cycle and contributes to the formation of glutamate in neurons (Brekke et al., 2012). Thus, it is tempting to hypothesize, but remains to be proven that increased PPP activity following TBI reflects a response by injured cells to combat oxidative/nitrosative stress and/or provide additional substrates for oxidative metabolism.

### Oxidative metabolism following TBI

As previously described, mitochondrial dysfunction is thought to play a key role in the pathophysiology of TBI. Studies using cytochrome C oxidase histochemistry as a measure of oxidative phosphorylation on the mitochondrial membrane, show a diffuse decrease in staining throughout the injured hemisphere of both FPI (Hovda et al., 1991) and CCI (Moro and Sutton, 2010) injured adult rats. Moreover, measurements of mitochondrial respiration rates have shown TBI-induced reductions in mitochondrial state 3 respiratory rates in immature and adult rat experimental models and humans (Xiong et al., 1997; Verweij et al., 2000; Kilbaugh et al., 2011). Neuroprotective strategies targeting mitochondrial dysfunction such as cyclosporin A (or its analog), oxidative/nitrosative species scavengers, or alternative metabolic substrates to glucose have shown reduced cell death, improvements in mitochondrial function, and/or functional outcome (Fukushima et al., 2009; Moro and Sutton, 2010; Mustafa et al., 2010; Kilbaugh et al., 2011; Readnower et al., 2011; Singh et al., 2013). Mitochondrial dysfunction, specifically changes in the oxidative metabolism of metabolic fuels, can be measured using ^13^C NMR spectroscopy by determining the amount of ^13^C incorporation into glutamate and glutamine. However, oxidative metabolism in astrocytes and the specific contribution of glutamine to metabolic compartmentation is more accurately measured using acetate, a glial specific substrate (Waniewski and Martin, 1998; Lebon et al., 2002; Deelchand et al., 2009; Shen, 2013) or [2 ^13^C] glucose that preferentially labels glutamine via pyruvate carboxylase (PC).

Using an adult rat CCI injury model (Bartnik et al., 2005), the amount of ^13^C label incorporated into the glutamate C2, C3, and C4 isotopomers did not differ from naive, suggesting that oxidative metabolism and the activity of PDH in glutamatergic neurons is maintained in the injured cortex over the first 24 h after injury. In the same study, a significant increase in ^13^C labeling of the glutamine C3 isotopomer was detected at 3.5 h after injury. Since the specific contribution of glutamine labeling via oxidative metabolism in astrocytes is difficult to ascertain using [1, 2 ^13^C_2_] labeled glucose, this study could not clarify if the increased labeling of glutamine reflected the *de novo* synthesis of glutamine or increased glutamate uptake by astrocytes in response to injury. In support of the latter mechanism, increased glutamate metabolism via the astrocytic TCA cycle occurs when extracellular glutamate concentrations are increased (McKenna et al., 1996) and during ischemia (Haberg et al., 1998; Pascual et al., 1998). Also, excitotoxic injury in rats alters glutamate-glutamine cycle enzymes to favor increased glutamine synthesis (Ramonet et al., 2004). In contrast to CCI, adult rats with FPI showed reduced ^13^C labeling of all glutamate and glutamine isotopomers at 3.5 h post injury, indicating reduced oxidative metabolism in both neurons and astrocytes in the injured cortex (Bartnik et al., 2007). In this model, the ^13^C labeling of glutamate returned to non-injury levels by 24 h while reductions in glutamine labeling persisted. The divergent pattern of ^13^C labeling between these two injury models likely represents previously reported differences in the extent and severity of CMRglc changes in the two models (Yoshino et al., 1991; Sutton et al., 1994; Lee et al., 1999; Moore et al., 2000).

In contrast to what is observed in studies using adult models, a ^13^C NMR spectroscopy study of the injured immature rat brain found increased labeling of glutamate and glutamine C3 and C4 isotopomers at 5.5 and 6 h following CCI injury (Scafidi et al., 2009). Scafidi et al. (2009) proposed that there could be an accumulation of glutamate due to impaired glutamate entry into the mitochondria via reduced activity of the aspartate-glutamate carrier (McKenna et al., 2006; McKenna, 2007), or reduced glutamate oxidation to α-ketoglutarate via decreased activity of α-ketoglutarate dehydrogenase due to oxidative stress (Starkov et al., 2004). The delayed increase in labeling also suggests a delay in the metabolic response in the immature brain and highlight important developmental differences in the response to injury (Scafidi et al., 2009).

### Astrocyte metabolism and neuroglia metabolic compartmentation following TBI

Astrocytes show pronounced changes in gene expression, cellular hypertrophy and proliferation, in a degree relative to the severity of brain injury. Studies in both experimental and human brain injury have demonstrated the presence of reactive astrocytes (Bourke et al., 1980; Cortez et al., 1989; Castejón, 1998). Reactive astrocytes play dual roles following injury, one that may result in a detrimental increase in glutamate excitotoxicity or inflammation, the other being brain protection or repair (Laird et al., 2008). A transient down regulation of glutamate transporters GLT-1, GLT-1v, and GLAST on astrocytes after experimental (Rao et al., 1998, 2001; Yi and Hazell, 2006) and human TBI (van Landeghem et al., 2006; Beschorner et al., 2007) may well contribute to the injury process. However, ablation of reactive astrocytes following experimental CCI in transgenic mice resulted in greater loss of cortical tissue and inflammation, suggesting an essential protective role for astrocytes after TBI (Myer et al., 2006).

Determining the role that astrocytes play in the metabolic response to TBI is an important research direction. Astrocytes play a pivotal role in meeting the energy requirements of neurons through the glutamate-glutamine cycle that links the exchange of glutamate and glutamine between glutamatergic neurons and astrocytes (Van den Berg et al., 1969). Another proposed mechanism of metabolite trafficking between these cells is the lactate shuttle, where astrocytes preferentially metabolize glucose via glycolysis and transfer lactate to neurons during high metabolic demand (Magistretti and Pellerin, 1999; Bouzier-Sore et al., 2002; Pellerin et al., 2007), although yet to be proven and a topic of ongoing debate (Jolivet et al., 2010; Mangia et al., 2011). The net synthesis of glutamate in neurons also requires a compensatory flux of TCA cycle intermediates, notably glutamine from astrocytes (Schousboe et al., 1997), as neurons lack the capacity to generate TCA cycle intermediates. This net synthesis of TCA cycle intermediates, glutamate and glutamine depends upon the entry of pyruvate, via an anaplerotic pathway, into the TCA cycle. In the brain this is exclusively achieved by PC, an astrocyte specific enzyme (Yu et al., 1983; Shank et al., 1985). Numerous *in vitro* studies have shown that astrocytes supply TCA cycle substrates to neurons during periods of glucose and/or oxygen deprivation (Hertz, 2003; Bambrick et al., 2004; Peng et al., 2007), suggesting that astrocytes may play an even greater nutritional role for neurons in the injured state. Given the essential role of neuroglia metabolic coupling in normal brain, a greater appreciation of the effect of TBI on metabolic coupling is an important and necessary contribution to understanding the metabolic response to TBI.

**Figure 2:**
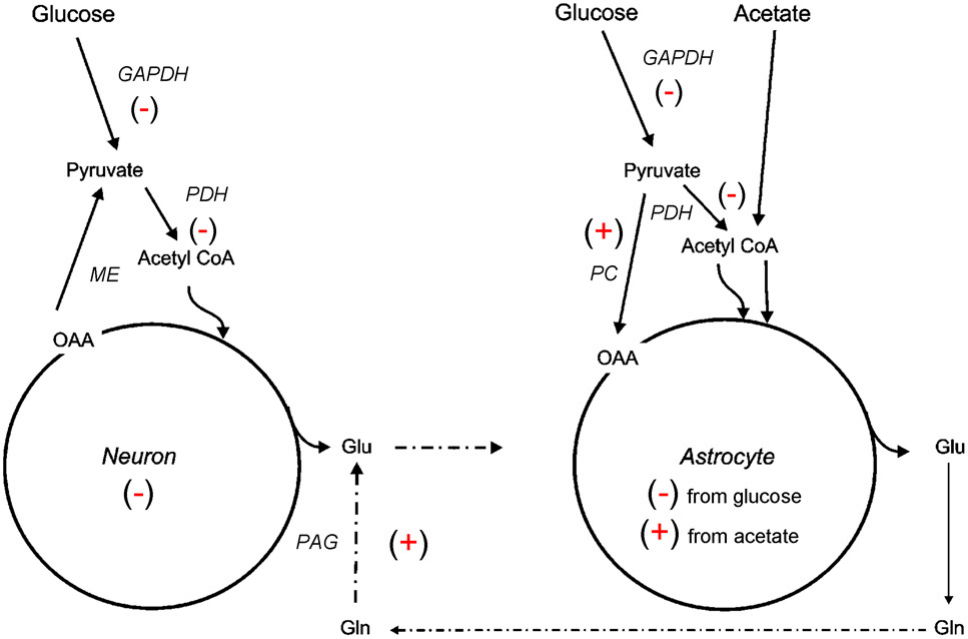
**Simplified illustration of changes in neuroglial metabolic coupling following FPI as determined using ^13^C labeled glucose and acetate.** Findings show reduced (−) glucose metabolism in both neuron and astrocyte metabolic compartments, possibly due to reduced activity of GAPDH and/or PDH. The capacity for oxidative metabolism was retained (^+^) in the astrocyte compartment as ^13^C labeling was detected in glutamine isotopomers resulting from acetate metabolism via the TCA cycle and PC. Labeling of glutamate from ^13^C acetate indicates continued activity (^+^) of the glutamate-glutamine cycle and phosphate activated glutaminase (PAG). These findings could be interpreted to mean that astrocytes have a supportive metabolic role to neurons following TBI.

The ^13^C NMR studies detailed in section Glycolysis and PPP Metabolism Following TBI suggest that neuroglia metabolic coupling is altered in two different rat models of TBI. To more clearly define the contribution of this metabolic coupling over the hypometabolic period, a ^13^C NMR spectroscopy study using [1 ^13^C] glucose, which is consumed in both neuronal and glial compartments, and [1, 2 ^13^C_2_] acetate, which is metabolized solely within the glial compartment, was undertaken using an adult rat FPI model (Bartnik-Olson et al., 2010). Figure 2 illustrates the metabolic alterations to neuronal and astrocyte metabolism determined using this strategy. Similar to previous findings, decreased ^13^C labeling of all glutamate isotopomers from the metabolism of glucose was observed early post-injury, but recovered over time, indicating that injury-induced decreases in the oxidative metabolism of glucose in neurons is consistent with the time course of reduced CMRglc following FPI (Yoshino et al., 1991; Moore et al., 2000). Although the ^13^C labeling of glutamine C4 from glucose in the first turn of the astrocyte TCA cycle was reduced, the labeling of glutamine C2 and C3 remained unchanged, indicating that the metabolism of glucose via PC was unaffected by FPI. In addition, the incorporation of ^13^C label from acetate into glutamine and glutamate C4 was maintained, indicating that oxidative metabolism in astrocytes and the functional activity of the glutamate-glutamine cycle were preserved during the hypometabolic period following FPI. ^13^C labeling of glutamine from ^13^C acetate was also demonstrated following human TBI using microdialysis samples and ^13^C NMR spectroscopy (Gallagher et al., 2009), although glutamate labeling was seen in only a few patients. It is important to note that acetate enters the astrocyte TCA cycle as acetyl CoA, bypassing any dysfunction in glycolysis or at the level of PDH, which may relate to the ability of an acetate precursor to improve ATP and improve motor performance after CCI (Arun et al., 2010).

### Limitations and future directions

The studies reviewed above highlight alterations to a number of key metabolic processes during the period of metabolic depression following experimental TBI. Although these studies are valuable in their contributions linking the period of metabolic depression to qualitative changes in a number of metabolic processes, they are limited by their descriptive nature. *In vivo* metabolic reactions are dynamic and future studies making use of mathematical models to extract quantitative flux rates would vastly improve our understanding of TBI-induced changes in neuroglia compartmentation and neurotransmission. Moreover, clinical (human) studies using dynamic ^13^C NMR spectroscopy is a logical next step in advancing our understanding brain function after TBI.

One goal of future animal and clinical ^13^C studies should be to understand the cellular basis of metabolic alterations following TBI. It is important to establish how individual cell types respond to TBI. For example, studies employing compartment specific labels (singly or in combination) could delineate a cell-type specific preference for a metabolic fuel that would preferentially enhance outcome. In addition, future studies of metabolic flux during the acute period of hyperglycolysis could provide direct evidence of the metabolic forces (increased neurotransmission and/or energetics) driving this need. Moreover, comparisons between findings from the acute period of hyperglycolysis and the period of metabolic depression could establish key time points and potential targets for metabolic intervention.

## Conclusion

TBI induces multiple primary and secondary injury mechanisms that can impact the supply of fuels and/or alter the functions of metabolic enzymes and proteins which can lead to deficits in energy availability. ^13^C NMR spectroscopy can be utilized to probe multiple aspects of the metabolic response to TBI, including changes in glycolysis, PPP activity, oxidative metabolism, and neuroglial metabolic compartmentation. As illustrated in the materials reviewed above, numerous experimental treatments that improve cerebral metabolism, reduce neuronal injury, and improve functional outcomes after TBI are currently being investigated, and future studies using ^13^C NMR spectroscopy to evaluate the metabolic responses to such treatments should provide valuable insights into the mechanisms of actions.

## Author contributions

Dr's. Bartnik-Olson and Sutton prepared the initial draft of this manuscript, Dr's. Harris and Shijo contributed additions and edits to the final versions of the manuscript.

### Conflict of interest statement

The authors declare that the research was conducted in the absence of any commercial or financial relationships that could be construed as a potential conflict of interest.

## Abbreviations

ATP: adenosine triphosphate
Ca^++^: calcium
CCI: controlled cortical impact
CMRglc: cerebral metabolic rate of glucose
FPI: fluid percussion injury
G6PDH: glyceraldehyde-6-phosphate dehydrogenase
GAPDH: glyceraldehyde-3-phosphate dehydrogenase
GC-MS: gas chromatography-mass spectrometry
GLAST: glutamate-aspartate transporter
GLT-1: glutamate transporter 1
GLT-1v: glutamate transporter 1 variant
NAD^+^: nicotinamide adenine dinucleotide phosphate
NADP^+^: nicotinamide adenine dinucleotide phosphate
NMR: nuclear magnetic resonance spectroscopy
PARP: poly(ADP) ribose polymerases
PC: pyruvate carboxylase
PDH: pyruvate dehydrogenase
PPP: pentose phosphate pathway
TCA: tricarboxylic acid cycle
TBI: traumatic brain injury.
